# 

**Published:** 1882-07

**Authors:** 


### AMERICAN MEDICAL ASSOCIATION.

The thirty-third annual meeting of the American Medical Association will be held at St. Paul, Minnesota, commencing Tuesday, June 6th, and lasting four days. A section in dentistry was formed in this Association at its last meeting, and medically educated dentists were recognized as specialists in medical science. All medical men of the regular school, practicing the specialty of dental surgery, are most cordially invited to procure credentials from their local medical societies, and join us at St. Paul. All railroads furnish reduced rates to those members wishing to attend. A member desiring to read a paper before any section, should forward the paper, or its title and length (not to exceed twenty minutes in reading) to the chairman of the Committee of Arrangements at least one month before.

### EDITORIAL.

### THE AMERICAN DENTAL ASSOCIATION.

The approaching meeting of this body, to be held in Cincinnati on the first of August, will be the most important one ever held—not only for the body itself, but for the profession. The latter now occupies a position of greater prominence and interest than at any time hitherto; it is more highly appreciated by the public.

By the action of the American Medical Association, and the International Medical Congress, and quite a number of State and local medical societies, the dental profession is placed in a position it never before occupied.

The action of quite a number of medical colleges in the establishment in each of a regular professorship of oral science and surgery, and the organization of several dental colleges—all within the last two or three years—are facts of special significance that should not be overlooked nor lightly esteemed. In them is much of promise for the future. They involve an increased responsibility for those who take part in our professional movements and activities.

The increase of our periodical literature has been quite appreciable within a few years; this arises from a demand which is based upon real progress. There are more readers, students, and investigators in the profession than ever before. Now, in view of all these things, does not a great responsibility devolve upon this body, which, in a large measure is regarded as the representative body of the dental profession.

It is desirable and important that the American Dental Association should recognize the trend of events that are taking place, and shape its proceedings in harmony with the things that make for progress and improvement, and endeavor to arrest, or at least to modify, whatever is manifestly adverse to public welfare and professional interest.

It is to be hoped that personal interests and enterprises will be kept in abeyance, or be made subservient to the public interest.

May fruitless personal wrangles be thrown into the dark corner, and kept there.

Three great divisions of work call for, and should receive, the earnest attention of the Association, viz., its science, its art, and its educational interests. The work upon these three branches should be better done than ever before. Let every one come with his heart warm and his head and hands full with that which shall make for peace and prosperity.

### GOING ABROAD.

Dr. Alphonso Taft, D.D.S., of Cincinnati, sailed June 29th with the intention of taking up his residence, for a time at least, in Berlin, Germany. His expectation is to take up the practice of his profession in connection with Dr. A. Sylvester, D.D.S., a gentleman of recognized ability, who has been in successful practice in that city for quite a number of years.

Those who place themselves in the professional charge of these gentlemen will receive careful and prompt attention, and the exercise of a good degree of skill and experience.

### AMERICAN DENTAL ASSOCIATION.

The twenty-second annual session of the American Dental Association will be held at Cincinnati, commencing Tuesday, August 1, 1882.

GEO. H. CUSHING,
Recording Secretary.

### PENNSYLVANIA STATE DENTAL SOCIETY.

The fourteenth annual meeting of this society will be held in Williamsport, Pa., July 26th, at 10 o'clock A.M. Session will continue for three days.

Dentists having special cases available, either in operative, mechanical or surgical clinics, will please inform the committee at once.

Persons having inventions or improvements of interest to the profession, will please notify the committee, who will make arrangements to have them properly exhibited. A full programme will be published hereafter.

G. W. KLUMP,

Chairman of Committee.

WILLIAMSPORT, Pa.

### CONTENTS.

### COMMUNICATIONS.

### THE DENTAL REGISTER,

A MONTHLY MAGAZINE DEVOTED TO THE INTERESTS OF THE

### DENTAL PROFESSION.

*Terms Reduced to $2.00 Per Year. Single Copies, 25 Cents.*

### EDITED BY PROF. J. TAFT, D.D.S.

—AND—

### *Published by SPENCER & CROCKER, Ohio Dental Depot.*

The volume commences in January and closes in December.

The REGISTER being issued monthly is always fresh, therefore Indispensable to the practitioner who desires to keep posted up with the new inventions and improvements which are daily developed. Neither expense nor effort will be spared to give value and variety to its contents. Articles will be illustrated with well executed engravings whenever they will add to the interest or assist to explanation.

Its extensive circulation and frequency of issue make it one of the BEST DENTAL ADVERTISING MEDIUMS in the United States.

All subscriptions must begin with January or July.

Local or special notices, five lines or less, will be inserted for $3 each insertion; over five lines, 50 cts. per line.

All advertising bills payable in advance, or at furthest, after the issue of the first number containing the advertisement All transient advertisements to be paid for in advance.

☞CONTRIBUTIONS SOLICITED.

Exclusively Editorial Communications should be addressed to the Editor.

The latest number of the REGISTER may be ordered with or without other goods at any time, and all letters should be addressed to

**SPENCER & CROCKER, Ohio Dental Depot, Cincinnati, Ohio.**

